# The 5-Aminolevulinic Acid (5-ALA) Supplement Enhances PSII Photochemical Activity and Antioxidant Activity in the Late Growth Promotion of *Pseudostellaria heterophylla*

**DOI:** 10.3390/plants11223035

**Published:** 2022-11-10

**Authors:** Julin Ma, Meng Sun, Lingling Qiu, Yinfeng Xie, Yingli Ma, Wenchao Liang

**Affiliations:** 1 Co-Innovation Center for Sustainable Forestry in Southern China, College of Biology and the Environment, Nanjing Forestry University, Nanjing 210037, China; 2 Institute of Pomology, Jiangsu Academy of Agricultural Sciences, Jiangsu Key Laboratory for Horticultural Crop Genetic Improvement, Nanjing 210014, China

**Keywords:** *Pseudostellaria heterophylla*, 5-aminolevulinic acid, antioxidant enzyme, leaf photosynthesis, chlorophyll fluorescence parameters

## Abstract

This study focused on the physiological regulation and mechanism of exogenous 5-aminolevulinic acid (5-ALA) in the late growth of *P. heterophylla*. In the middle of May, different concentrations of 5-ALA (0, 10, 20, 50 mg·L^−1^) were sprayed on the leaves. The effects of 5-ALA on tuberous root growth, antioxidant enzyme system, gas exchange, photosynthetic pigment contents and photosynthetic characteristics were measured from 23 May to 13 June. A concentration of 20 mg·L^−1^ of 5-ALA led to a significant increase in the yield of fresh root and biomass allocation at 38.12% and 25.07%, respectively, in comparation with the control (0 mg·L^−1^). The moderate concentration of 5-ALA statistically stimulated antioxidation activities. 5-ALA treatment enhanced photosynthetic activity and reduced photodamage. Compared to the control, there were increases in the chlorophyll fluorescence parameters of *P. heterophylla* under 5-ALA treatment. Moreover, 20 mg·L^−1^ of 5-ALA significantly changed the kinetic parameters of fluorescence. It enhanced the light absorption and distribution efficiency of PSII and the activities of leaves, resulting in alleviating photoinhibition by the excess excitation energy. The correlation indicated that there was a significant positive correlation between the yield of tuberous roots and biomass allocation, P_n_ and catalase (CAT), and a negative correlation between the yield of tuberous roots and malondialdehyde (MDA). The appropriate 5-ALA concentration in the late growth stage of *P. heterophylla* effectively enhanced the net photosynthetic capacity, mainly resulting from the enhancement of PSII photochemical activity to promote the increases in excitation energy absorption, capture and electron transfer efficiency of the leaves. Finally, 5-ALA treatment can increase the photochemical activity of PSII in the whole leaf and ultimately delay the senescence of *P. heterophylla.*

## 1. Introduction

*Pseudostellaria heterophylla* is a dry tuberous root of the Caryophyllaceae family [1] and a traditional Chinese herb with the effect of nourishing the lungs and the qi to invigorate the spleen. It has a long history as a medicinal plant with proven effects and is a common clinical herb. The Ministry of Health has included it in the list of Chinese herbs that can be used in health foods. *P. heterophylla* has polysaccharides, saponins, flavonoids, cyclic peptides, amino acids and microelements from the tuberous roots [2], which together constitute its qualities and pharmacological functions such as reducing fatigue, protecting the heart muscle, improving immunity and antitumor activity [3]. It is widely used for medical purposes, cosmetics and dietary supplements, so there is an increasingly widespread demand for production. *P. heterophylla* is cold and high-temperature resistant and negatively affected by high light intensity and thus should be planted in a cool and humid environment [4]. In late spring and early summer, when the temperature reaches above 30 °C, the above-ground stem and leaves stop growing, and then the underground part enters into dormancy, which leads to a rapid decay of photosynthetic capacity, a shortening of the effective photosynthetic period and a decrease in yield. Therefore, it is critical to know how to enhance the physiological function of *P. heterophylla* in the late stage of growth and prolong the growth period to improve the yield of tuberous roots.

5-Aminolevulinic acid (5-ALA) is a promising chemical molecule found in a wide variety of organisms, including bacteria, algae, plants and animals. It is a key precursor for the synthesis of all porphyrin compounds (chlorophyll, haemoglobin, haematoxylin, vitamin B12 and phytochrome choline) [5]. It plays an important role in plant growth and development and can effectively improve the photosynthetic capacity of plants under adversity [6]. Yang Ni et al. [7] showed that 5-ALA treatment could significantly increase the pigment content, alleviate PSII photodamage and enhance the photosynthetic capacity of tea trees under drought stress. In recent years, there have been more studies on 5-ALA all over the world about the physiological aspects of plant photosynthesis, low-temperature resistance and salt tolerance [8] on vegetables and crops, for example, pakchoi [9], wheat [10] and potato [11]. However, little research has been reported about the effects of 5-ALA on photosynthesis and the regulation of tuberous roots in medicinal plants. Therefore, this study substantially contributes to improving our scientific understanding of different quality concentrations of 5-ALA responses in the mitigation of senescence in the late growth stage and the growth of the root tubers of *P. heterophylla*.

## 2. Results

### 2.1. Effects of 5-ALA on the Growth of Tuberous Roots

Table 1 shows the changes in the root length, root diameter, fresh root yield per unit area, aerial part dry weight, underground part dry weight, single plant biomass and root-shoot ratio under different 5-ALA concentrations. A concentration of 20 mg·L^−1^ of 5-ALA had the best effect on tuber length, fresh root yield, biomass allocation and root-shoot ratio, with significant increases of 29.86%, 38.12%, 25.07% and 44.74%, compared with the control (0 mg·L^−1^), respectively. The results indicated that 20 mg·L^−1^ of 5-ALA had a significant effect (*p* < 0.05) on the accumulation of tuberous root biomass and also facilitated the transfer of nutrients from the above-ground to the below-ground part, which promoted the yield of *P. heterophylla*.

### 2.2. Effects of 5-ALA on Antioxidant Enzyme Activity

**Figure 1:**
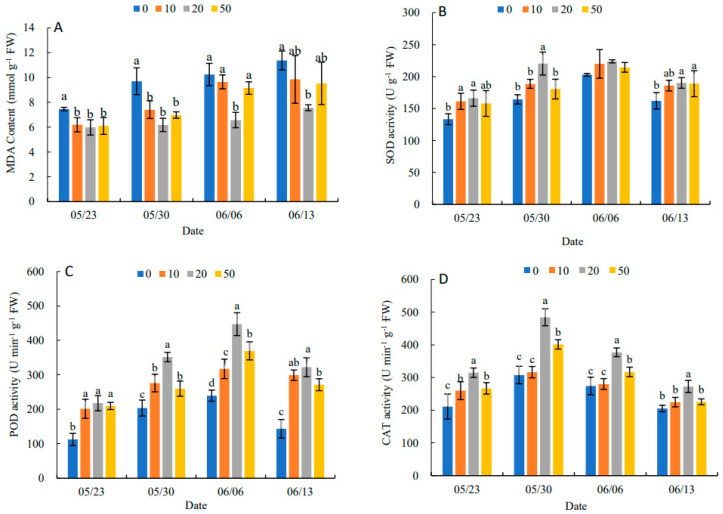
Effect of different concentrations of ALA on MDA content (**A**), SOD activity (**B**), POD activity (**C**) and CAT activity (**D**) of *P. heterophylla* leaves from 23 May to 13 June. Data show the mean with standard deviation bar of three replicates from samples. Different letters indicate a significant difference at 0.05 level between different treatments.

In Figure 1A, the MDA content in leaves showed an increasing trend during the experimental period. The different concentrations of 5-ALA had different inhibition of MDA content. A concentration of 20 mg·L^−1^of 5-ALA had the most obvious inhibitory effect on MDA content at different sampling points. It decreased by 19.84%, 36.25%, 35.81% and 33.48% compared with the control, respectively. This indicated that 5-ALA could effectively alleviate the intensification of membrane lipid peroxidation and reduce the structural damage of cell membranes in the leaves. In Figure 1B, compared with the control, all treatments increased the SOD activity in leaves to alleviate the decline in SOD activity at the later stage of the experiment, particularly the 20 mg·L^−1^ treatment had the best effect on SOD activity. The trend of POD activity was similar to that of SOD (Figure 1C). The results (Figure 1D) showed that three 5-ALA treatments reached peak values on 30 May, and CAT activities in treatments followed 20 mg·L^−1^ > 50 mg·L^−1^ > 10 mg·L^−1^ > 0 mg·L^−1^ at each time point. The results showed that SOD, POD and CAT activities showed an increasing and then decreasing trend under 5-ALA treatment. Thus, the three antioxidant enzymes had synergistic effects on effectively scavenging excessive free radicals and enhancing the antioxidant activity of leaves.

### 2.3. Effects on Photosynthetic Pigments in the Leaves

**Figure 2:**
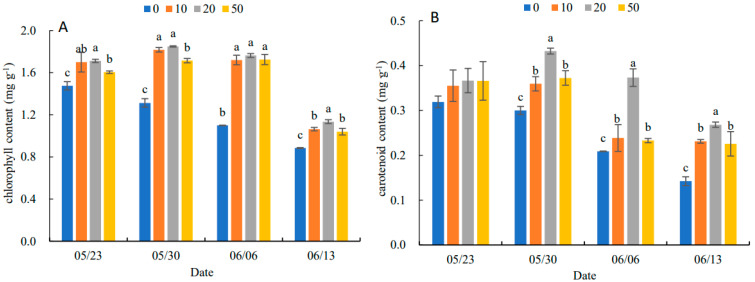
Effects of different concentrations of 5-ALA on chlorophyll content (**A**) and carotenoid content (**B**) of *P. heterophylla* leaves during senescence from 23 May to 13 June. Data show the mean with standard deviation bar of three replicates from samples. Different letters indicate a significant difference at 0.05 level between different treatments.

In Figure 2, the chlorophyll content and carotenoid content of leaves increased and then decreased with the increase in 5-ALA concentration. The 20 mg·L^−1^ treatment was the best concentration for the accumulation of pigment contents. On 13 June, the chlorophyll content of the different treatments was higher than that of the control (0 mg·L^−1^) by 8.74%, 30.55%, 56.96% and 17.57%, respectively. The carotenoid content of the different treatments increased by 15.63%, 43.34%, 76.19% and 92.86%, compared to the control, respectively.

### 2.4. Effects on the Dynamic Parameters of Daily Photosynthetic Changes

**Figure 3:**
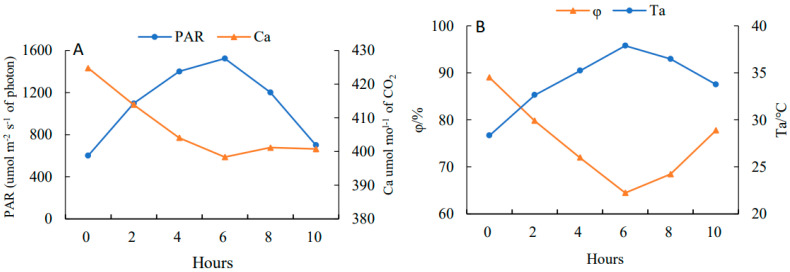
Diurnal variations of photosynthetically active radiation (PAR) (**A**), atmospheric CO_2_ concentration (Ca) (**A**), atmospheric temperature (Ta) (**B**) and atmospheric humidity (φ) (**B**) in 10 h (from 7:00 to 17:00).

On a clear day, PAR (Figure 3A) showed a rising and then falling trend with a daily average value of 1287.02 μmol·m^−2^·s^−1^ and reached the peak at 13:00 (1723.37 μmol·m^−2^·s^−1^), while Ca (Figure 3A) showed a falling and then rising trend with a daily average value of 607.16 μmol·mol^−1^ and had a minimum value at 13:00 (598.36 μmol·mol^−1^). The trend of Ta (Figure 3B) was consistent with PAR, having a daily average of 34.88 °C with a peak at 13:00 (37.88 °C). The trend of φ (Figure 3B) was similar to Ca, with a daily average of 75.24% and a trough at 13:00 (64.47%).

**Figure 4:**
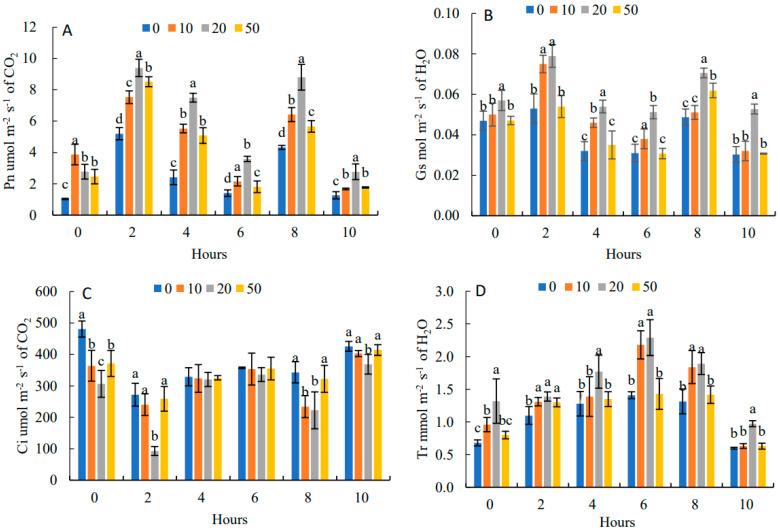
Effects of 5-ALA on diurnal changes of net photosynthetic rate (Pn) (**A**), stomatal conductance (Gs) (**B**), intercellular carbon dioxide concentration (Ci) (**C**) and transpiration rate (Tr) (**D**) of *P. heterophylla* in 10 h (from 7:00 to 17:00). Data show the mean with standard deviation bar of three replicates from samples. Different letters indicate a significant difference at 0.05 level between different treatments.

In Figure 4A, the diurnal variation of Pn was a bimodal tendency with two peaks at 9:00 and 15:00, respectively, and the first peak was higher than the second peak. The daily average value of Pn was the highest under 20 mg·L^−1^, which was 85.62% higher than that of the control. It showed that an appropriate concentration of 5-ALA can effectively alleviate the photosynthetic noon break phenomenon. The daily trend of Gs (Figure 4B) and Pn were the same, showing “M” curves with the two peaks at 9:00 and 15:00. The daily change in Gs under 20 mg·L^−1^ treatment was the highest, which was 50.0% higher than that of the control. In Figure 4C, the daily trend of Ci was opposite to that of Pn, showing a “W” curve with two troughs at 9:00 and 15:00. The daily mean values of Ci in each of the 5-ALA treatments were lower than that of the control by 13.73%, 24.37% and 6.97%, respectively.

Figure 4D shows that the diurnal variation of Tr under different 5-ALA treatments increased and then decreased with a peak at 13:00. Tr presented an increasing and then decreasing trend. Under the 20 mg·L^−1^ treatment, *T*r had the maximum value, which was higher than that of the control by 22.69%.

### 2.5. Effects on Dynamic Changes of Chlorophyll Fluorescence Parameters in Leaves

**Figure 5:**
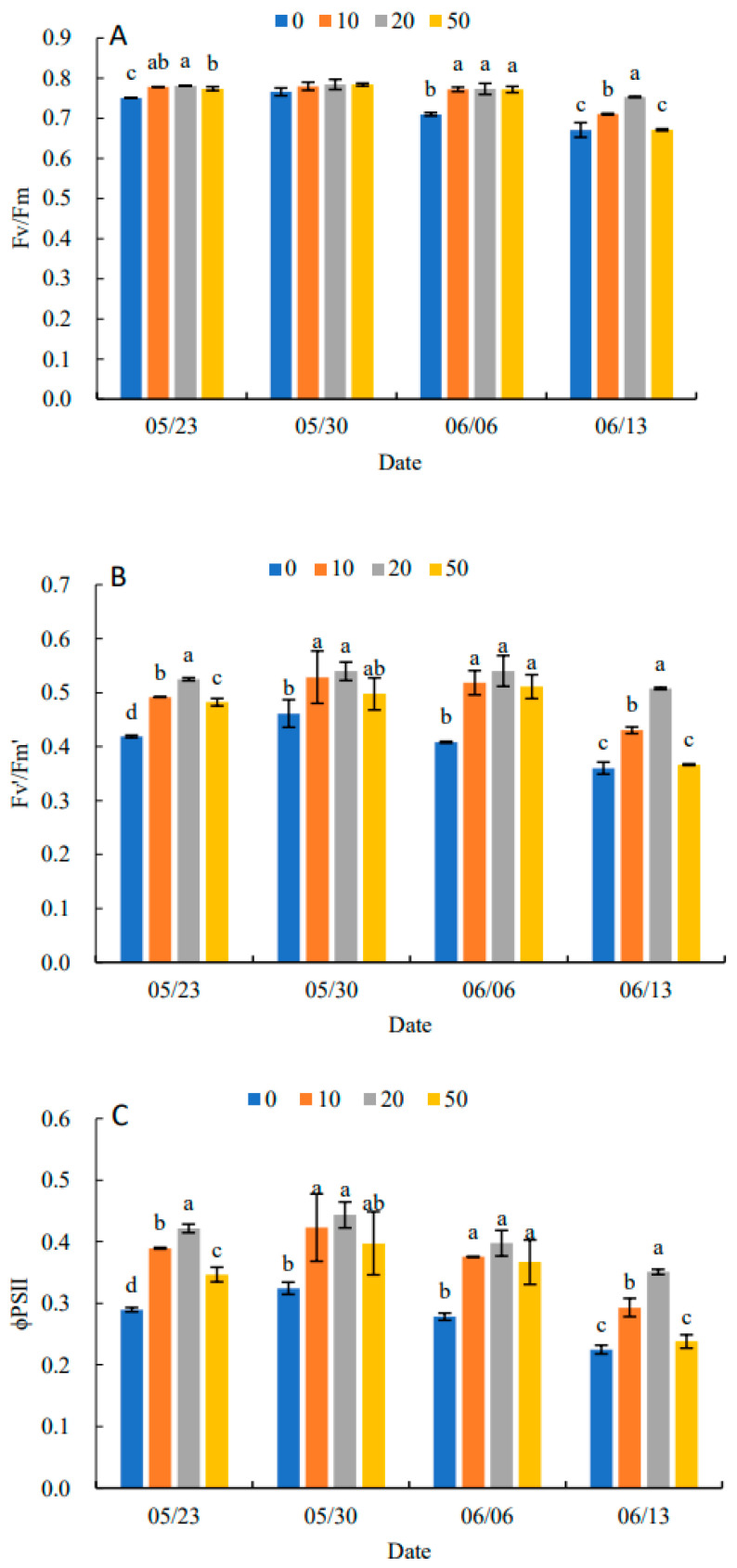

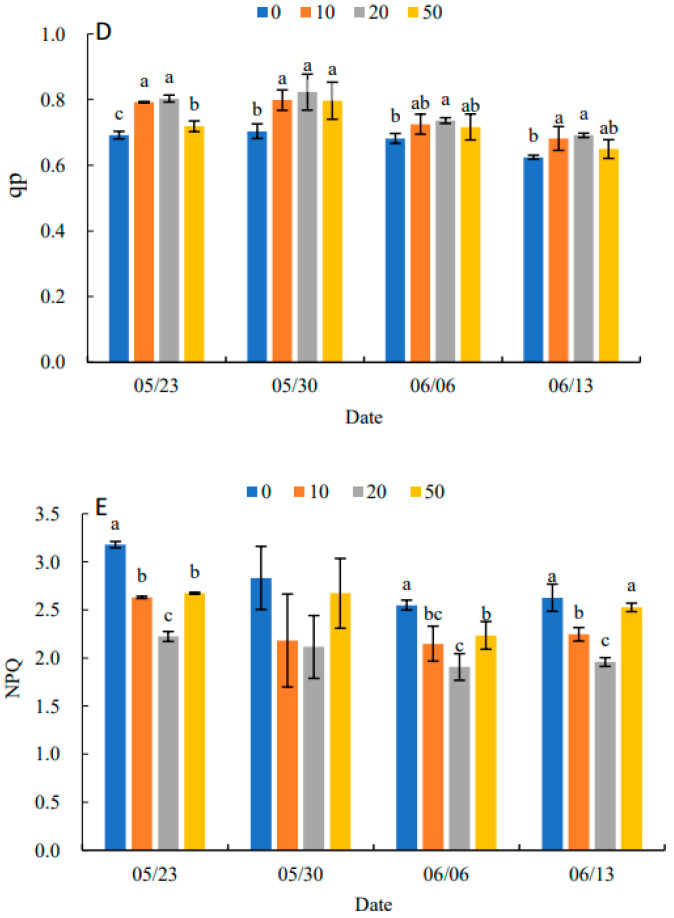
Effects of different concentrations of 5-ALA on the maximal efficiency of PSII photochemistry (Fv/Fm) (**A**), the effective efficiency of PSII photochemistry (Fv′/Fm′) (**B**), actual PSII efficiency (Φ_PSII_) (**C**), photochemical quenching (qP) (**D**) and non-photochemical (NPQ) (**E**) in *P. heterophylla* from 23 May to 13 June. Different letters indicate a significant difference at 0.05 level between different treatments.

Fv/Fm under all treatments showed an increasing and then decreasing trend in Figure 5A. Compared with the control, 5-ALA increased Fv/Fm, particularly under the 20 mg·L^−1^ treatment. When there was no significant difference between treatments, 5-ALA treatments were significantly higher than the control, except for 30 May. On 13 June, the 20 mg·L^−1^ treatment was 11.94% higher than the control. In Figure 5B–D, Fv′/Fm′, ΦPSII and qP in each treatment increased and then decreased. They reached their maximum value on 30 May, and Fv′/Fm′, ΦPSII and qP were higher than the control by 17.39%, 37.50% and 17.14%, respectively. The results in Figure 5E indicated that NPQ in each treatment showed a decreasing and then increasing trend, and all reached their minimum values on 6 June. Therefore, the plant growth regulator treatments were effective in increasing Fv/Fm, Fv′/Fm′, ΦPSII and qP and decreasing the NPQ of senescing leaves at the late stage of growth. The 20 mg·L^−1^ treatment had the most prominent effect on *P. heterophylla* leaves.

### 2.6. Effects on PSII Energy Partitioning and Specific Activity Parameters

A comparison of the quantum yield and energy partitioning ratio of the PSII of the control and the 20 mg·L^−1^ treatment was used to further explore the mechanism of 5-ALA enhancing the photochemical efficiency of PSII in *P. heterophylla*. In Table 2, φPo, ψo and φEo under the 20 mg·L^−1^ treatment were higher than the control by 21.67%, 15.79% and 39.13, respectively, while the quantum ratio of heat dissipation (φDo) was significantly lower than that of the control. ABS/RC, TRo/RC, ETo/RC and DIo/RC indicated the absorption, capture, transfer and dissipation of energy per unit area of the reaction center. ABS/CSm, TRo/CSm and ETo/CSm reflected the absorption, capture and conversion of light energy by the photosynthetic organs of the plants [12]. The results showed that there were no statistical differences between ABS/RC, TRo/RC, ETo/RC and DIo/RC in the control, while ABS/RC, TRo/RC and DIo/RC decreased, and ETo/RC increased under the 20 mg·L^−1^ treatment. Thus, the 20 mg·L^−1^ treatment increased the light energy transferred per unit area.

### 2.7. Correlation Analysis

The results of the correlation analysis showed that the yield of *P. heterophylla* was significantly positively correlated with single plant biomass, Pn and CAT, while it was significantly negatively correlated with MDA. Pn was significantly positively correlated with φPSII and qP but significantly negatively correlated with Ci and NPQ. Gs was significantly negatively correlated with Ci and NPQ. Finally, SOD was significantly positively correlated with POD, and MDA was significantly negatively correlated with CAT (Table 3).

## 3. Discussion

*P. heterophylla* is cold tolerant and heat sensitive, preferring shade and humidity, and is mostly found in wetlands under forests or thickets [13]. Due to environmental conditions such as high temperature, strong light and drought, *P. heterophylla* cultivation is more susceptible to abiotic stresses, and early physiological decline occurs in the later stages of growth, resulting in the later growth and yield of tuberous roots [14]. As with the results of the pre-experiment, the most significant increases in root yield, individual plant biomass, above-ground dry weight, below-ground dry weight and root-to-shoot ratio were observed under the 20 mg·L^−1^ 5-ALA treatment. Under severe heat stress, ROS dysregulation and accumulation in excess occur along with membrane lipid peroxidation and protein oxidation. The content of malondialdehyde (MDA), a product of membrane lipid peroxidation, increases, resulting in damage to the structure and function of the membrane, cell senescence and death, and growth inhibition [15]. Our study indicated that 5-ALA alleviated the increase in the MDA content at the late growth stage of *P. heterophylla*, which was consistent with the results of Sunman MSE et al. [10]. SOD, POD and CAT, as the main protective enzymes for scavenging reactive oxygen species, play important regulatory roles in maintaining the balance of reactive oxygen species metabolism in plants [16]. In this study, 5-ALA significantly increased the activities of SOD, POD and CAT in the late stage of growth, demonstrating that 5-ALA enhanced the antioxidant capacity of *P. heterophylla* by SOD, POD and CAT to protect the photosynthetic mechanism and maintain photosynthetic activity, promote growth and increase the yield of *P. heterophylla*. It also was supported by the negative correlation of yield and biomass with MDA content and the positive correlation with CAT activity in *P. heterophylla*. The regulation of CAT activity may play a major role in the regulation of antioxidant activity by 5-ALA because CAT is a binding enzyme with iron porphyrin as a cofactor for the biodefense system [17]. 5-ALA, as a precursor for the biosynthesis of porphyrins such as ferrous heme, heme (iron porphyrin) and vitamin B12, facilitates the synthesis of CAT with iron porphyrin as a cofactor and POD with ferrous heme as a cofactor [5].

Photosynthesis is the physiological basis of plant growth and yield and is also an important indicator of plant senescence and resistance to stresses [18]. The present study showed that the MDA content of leaves increased, while the chlorophyll and carotenoid contents gradually decreased at the end of spring and the beginning of summer, indicating that the physiological function of the leaves gradually declined. This suggested that 5-ALA alleviated the changes in MDA and the chlorophyll and carotenoid contents for the improvement of the physiological function of the leaves. Meanwhile, the enhanced activities of SOD, POD and CAT were related to the enhancement of antioxidant enzyme activities in reducing leaf senescence in the late stage of growth. Additionally, it was beneficial to the damage of chloroplast structure and function caused by membrane lipid peroxidation and the acceleration of the photosynthetic performance [19]. The alleviation of leaf senescence facilitated the extension of the photosynthetic effective stage and promoted the synthesis and accumulation of organic matter and growth. In addition, the results of the correlation analysis showed that the yield and single plant biomass were positively correlated with antioxidant activity (Table 3). POD and other enzymes were based on heme, and 5-ALA is a precursor of heme biosynthesis. Thus, 5-ALA may be converted to heme for the increase in the antioxidant enzyme activity in the leaves of *P. heterophylla* [20]. This mechanism should be further investigated in the future.

The daily variation of photosynthetic gas exchange parameters was not only a key indicator of the daily production capacity of photosynthesis but also the sustainable capacity of plant physiological metabolism and material accumulation [21]. In this study, the Pn of the control showed a typical bimodal variation, and the phenomenon of photosynthetic “noon-break” was obvious. Under the 20 mg·L^−1^ 5-ALA treatment, the diurnal trend of Pn presented a bimodal variation, but the treatment increased the daily mean, the peak and valley values, and the “noon break” phenomenon was effectively alleviated. This study was consistent with Farquhar et al. [22]. Under 5-ALA treatment, the enhancements of Pn and Gs were accompanied by a decrease in Ci after, which indicated that 5-ALA enhanced the photosynthetic activity of mesophyll cells by improving the non-stomatal limitation to increase the net photosynthetic capacity [8].

Chlorophyll fluorescence was considered a prospective marker of photosynthetic activity and an important indicator of carbon cycling in plants, which can further analyze the mechanism of 5-ALA to enhance photosynthetic performance in *P. heterophylla* [23]. PSII is the most sensitive component of photosynthetic organs [24]. It is also the primary and main site where photoinhibition occurs under environmental stresses such as high temperature and strong light [25]. Fv/Fm can reflect whether photoinhibition occurs in plants under dark adaptation. It was around 0.8 under non-adverse conditions and not easily affected by species [26]. In the later stages of growth, the Fv/Fm of the control continued to decline and was significantly lower than 0.75. The results indicated that PSII photoinhibition was enhanced with environmental stresses and leaf senescence in the later stages of growth, resulting in a significant decrease in the efficiency of excitation energy use (Fv′/Fm′ and ΦPSII). The results were further evidence that the growth characteristics of *P. heterophylla* were not tolerant to strong light and high temperatures. The increases in excess excitation energy increased the production of reactive oxygen species [27] and the destruction of photosynthetic mechanisms [28]. According to the trends in MDA and chlorophyll content in this study, it assumed that the increases in membrane lipid peroxidation and chloroplast pigment degradation in the later stages of growth were related to the increases in PSII photoinhibition and photodamage. Heat dissipation is an effective way for plants to resist photoinhibition, but NPQ in the control showed a decreasing trend, which may be a reflection of photo destruction induced by leaf senescence [29]. The 20 mg·L^−1^ 5-ALA treatment effectively alleviated the Fv/Fm, Fv′/Fm′, ΦPSII and qP, demonstrating that 5-ALA enhanced the photosynthetic performance at the later growth stage through the improvement of the photochemical efficiency of PSII. The enhanced photochemical efficiency was attributed to alleviating the photoinhibition and photodamage by the excess excitation energy.

φPo, ψo, φEo and φDo are the fluorescence parameters in relation to the energy partitioning ratio. They can more precisely reflect the absorption, conversion and dissipation of light energy than the activity parameters [30]. φPo indicated the ratio of the energy of electron transfer to the captured energy by the active reaction center and also reflected the maximum photochemical efficiency of PSII. In this study, the increase in φPo under 20 mg·L^−1^ 5-ALA stated that the PSII reaction center was disrupted, while ψo and φEo showed the efficiency of energy transferred from QA to QB downstream and the ratio of light energy absorption used for electron transfer after QA, respectively. The increases in ψo and φEo under the 20 mg·L^−1^ 5-ALA treatment indicated that the openness of the PSII reaction center increased and the ratio of energy used for the increase in electron transfer and QA reduction. Therefore, the increases in φPo, ψo and φEo and the decrease in φDo under the 20 mg·L^−1^ 5-ALA treatment demonstrated that 5-ALA adjusted the ratio of energy distribution in PSII reaction centers, increased the quantum ratio for electron transfer, and decreased the quantum ratio for heat dissipation. These results were consistent with the previous research that the increases in ABS/RC, TRo/RC and DIo/RC under adversity stresses may be due to a compensatory response following a decrease in the number of active reaction centers per unit leaf area [31]. *P. heterophylla,* under high temperatures and drought, used a defense system to decrease the photodamage induced by the accumulation of the excess excitation energy. PIabs not only reflected the primary photochemical quantum yield but also combined with the density of reaction centers and electron transfer between PSI and PSII could reflect the photosystem activity in light energy absorption and capture and electron transfer [32]. In this study, PIabs increased under the 20 mg·L^−1^ 5-ALA treatment, indicating that 5-ALA can improve the light energy conversion efficiency and the open ratio of PSII reaction centers, increase the primary light energy conversion efficiency and electron transfer activity of photosynthesis, and facilitate the leaf PSII light reaction. The results of the density parameter of the PSII reaction centers (RC/CSo) further indicated that ALA treatment not only enhanced the activity of PSII reaction centers per unit in *P. heterophylla* but also increased the number of reaction centers per unit area of the leaves. Thus, the 5-ALA treatment increased the photochemical activity of PSII in the whole leaf.

## 4. Method and Materials

### 4.1. Sites and Materials

This study was conducted at the Xiashu forestry farm in Xiashu, Jurong, Jiangsu Province, belonging to the Nanjing Forestry University. The experimental field was located at 31°59′ N, 119° E, which is considered a subtropical monsoon climate with 15.2 °C annual mean air temperature, 1104 mm average annual rainfall, 2018 h of sunshine on average annually, and a 229 d frost-free period. The planting soil is yellow-brown earth from weight loam to loamy soil. Plough layer soil (0–20 cm) includes 0.704 g kg^−1^ of total nitrogen, 0.146 g kg^−1^ of total phosphorus, 12.5 mg kg^−1^ of available phosphorus, 103.7 mg kg^−1^ of rapidly available potassium, and 7.39 g kg^−1^ of organic matter with a pH of 4.5–5.5.

### 4.2. Experimental Design and Treatment

*P. heterophylla* seedlings were sown at the end of November 2019. There were 5 ridges for ridge-planting with a length of 14 m, a width of 1 m, and a height of 25 cm. Five gullies were ditched on the ridge row with a spacing of 15 cm and a depth of 15 cm. Compound fertilizer and ash manure were sprinkled into the gullies. A seed spacing of 3–4 cm and a seeding rate of 100 gm^−2^ were used for drilling *P. heterophylla*. On 9 May and 16 May 2020, 5-ALA was sprayed on two sunny days. There were 4 different concentrations of 5-ALA (0, 10, 20, 50 mg·L^−1^) for the treatments based on pre-test results. Each treatment had 3 replicated blocks, and each block was 2 m^2^ (2 m × 1 m). In total, there were 12 blocks in the study. 5-ALA was sprayed onto the leaves with water drops and processed twice at an interval of one week. Treatments were implemented at 18:00, resulting from the easy decomposition of 5-ALA under exposed sunlight. After treatment, observation and physiological indicators were sampled and determined every week, and biomass was measured at harvest.

### 4.3. Measurement of Physiological Indicate

#### 4.3.1. Growth Index

Twenty *P. heterophylla* plants were randomly selected from each block. The samples were washed and dried on the surface, divided into above-ground and below-ground parts, and weighed for fresh weight. Fresh roots were placed in an oven at 105 °C for 15 min, dried at 60 °C to a constant weight, and the dry weight biomass of each part was weighed. Root growth indicators included tuber length (length from bud eye to tuber thinning), tuber diameter (diameter at the point of tuber expansion), and tuber biomass.

#### 4.3.2. Antioxidant Enzyme Activity

The modified thiobarbituric acid (TBA) method was for the determination of malondialdehyde (MDA) [33]. Superoxide dismutase (SOD) activity was determined by photochemical reduction with SOD-inhibited azotetrazolium (NBT), peroxidase activity (POD) by guaiacol, and catalase (CAT) activity by potassium permanganate titration [13].

#### 4.3.3. Photosynthetic Pigment and Diurnal Variation Parameters of Leaf Photosynthesis

Chlorophyll and carotenoid contents were determined using an acetone–ethanolic equal-mix extraction method [34]. The daily photosynthetic parameters were measured by a Li-6400R (Li-cor, Lincoln, NE, USA) portable photosynthesis meter in the third week after treatments. Measurements selected uniformly growing leaves with six replicates of each treatment at 2 h intervals from 7:00 to 17:00 (0 h to 10 h). The following indicators were measured: photosynthetically active radiation (PAR), atmospheric CO_2_ concentration (C_a_), air temperature (T_a_), relative air humidity (φ), net photosynthetic rate (P_n_), stomatal conductance (G_s_), transpiration rate (T_r_) and intercellular CO_2_ concentration (C_i_).

#### 4.3.4. Measurement of Dynamic Changes in ChlorophyII Fluorescence Parameters

The dynamic changes in chlorophyll fluorescence parameters were measured using the ChlorophyII Fluorescence Imager (CF Imager, Technologica, Dr K. Oxborough and Mr J. Bartington, of the Department of Biological Sciences, University of Essex, UK) at 15:00 on 23 May. Indicators included maximum photochemical efficiency of PSII (F_v_/F_m_), effective photochemical efficiency of PSII (F_v_′/F_m_′), actual photochemical efficiency (Φ_PSII_), photochemical quenching coefficient (qP) and non-photochemical quenching coefficient (NPQ). ChlorophyII fluorescence kinetic parameters were used by Handy PEA (Hansatech, Norfolk, UK), including the ratio of the energy transferred by the electron to the energy captured by the active reaction center (φPo), the probability of the captured exciton from electrons to other electron acceptors downstream of the primary acceptor QA in the electron transport chain (ψo), the quantum yield of electron transport (φEo), the quantum ratio of heat dissipation (φDo), light energy absorption per unit area (ABS/CSm), light energy capture per unit area (TRo/CSm), the quantum yield of electron transport per unit area (ETo/CSm), heat dissipation per unit area (DIo/CSm), light energy absorptionper reaction center (ABS/RC), light energy capture per reaction center (TRo/RC), heat dissipation per reaction center (DIo/RC), quantum yield of electron transport per reaction center (ETo/RC), the number of reaction centers per unit area (RC/CSo), and performance index of light energy absorption (PIabs) [35].

#### 4.3.5. Statistical Analysis

Data were collated, calculated and plotted by Excel 2019. SPSS 26 was used to analyze the data for the mean values, univariate ANOVA test and Pearson test (the correlation between variables). Data in the graphs are mean ± standard deviation. The significance level was *p* < 0.05.

## 5. Conclusions

In conclusion, a 20 mg·L^−1^ exogenous 5-ALA treatment significantly enhanced the antioxidant activity and photosynthetic performance of the leaves at the late stage of growth. It also effectively alleviated the photoinhibition and the physiological functions of the leaves under stress to extend the photosynthetic effective stage. Thus, it promoted growth and increased the yield of *P. heterophylla* tuberous roots. 5-ALA improved photosynthesis through the enhancement of the photochemical activity of PSII and the promotion of the excitation energy absorption and capture and the electron transfer efficiency of leaves. The alleviation of photoinhibition and photodamage may be related to the enhanced antioxidant capacity, pigment contents and utilization of excess excitation energy in *P. heterophylla* under 5-ALA treatment. The results of this study provide a theoretical and applied basis for the high-yielding cultivation of *P. heterophylla*.

## Figures and Tables

**Table 1:** Effects of different concentrations of 5-ALA on growth of *P. heterophylla* tuberous roots.

5-ALA Concentration (mg·L^−1^)	Tuberous Root		Fresh Root	Aerial Part Weight (g)	Underground Part Weight (g)	Biomass Allocation (g)	Root-Shoot Ratio
	Root Length (cm)	Root Diameter (mm)	Fresh Root Yield (gm^−2^)				
0	3.45 ± 0.47 c	4.78 ± 0.37 c	302.97 ± 7.34 c	1.99 ± 0.99 b	1.52 ± 0.05 c	3.51 ± 0.76 c	0.76 ± 0.06 b
10	3.89 ± 0.30 b	5.01 ± 0.39 b	357.04 ± 10.34 b	2.16 ± 0.08 a	1.80 ± 0.12 b	3.96 ± 0.83 b	0.83 ± 0.06 b
20	4.48 ± 0.33 a	6.35 ± 0.73 a	418.47 ± 15.66 a	2.19 ± 0.09 a	2.20 ± 0.10 a	4.39 ± 1.01 a	1.01 ± 0.07 a
50	3.82 ± 0.32 b	5.91 ± 0.41 b	368.04 ± 11.69 b	2.19 ± 0.11 a	1.86 ± 0.11 b	4.05 ± 085 b	0.85 ± 0.08 b

Data show the mean ± standard deviation of three replicates from samples. Different lowercase letters indicate a significant difference at 0.05 level between different treatments.

**Table 2:** Effects of ALA on PSII energy distribution and specific activity parameters of *P. heterophylla* at late growth stage.

	φPo	Ψo	φEo	φDo	ABS/RC	TRo/RC	ETo/RC
0	0.60 ± 0.04 a	0.38 ± 0.03 a	0.23 ± 0.01 a	0.41 ± 0.04 a	4.18 ± 0.68 b	2.47 ± 0.27 a	0.95 ± 0.16 a
20	0.73 ± 0.03 a	0.44 ± 0.04 a	0.32 ± 0.02 a	0.27 ± 0.03 b	3.21 ± 0.31 a	2.45 ± 0.31 a	1.04 ± 0.11 a
	DIo/RC	ABS/CSm	TRo/CSm	ETo/CSm	DIo/CSo	RC/CSo	PIabs
0	1.71 ± 0.41 a	1200.67 ± 376.97 a	705.33 ± 189.41 a	271.67 ± 84.89 a	495.33 ± 188.50 a	100.69 ± 2.67 a	0.23 ± 0.05 b
20	0.85 ± 0.02 b	1384.33 ± 179.27 a	1016 ± 153.91 a	450.33 ± 81.19 a	368.33 ± 42.06 a	95.31 ± 1.74 b	0.69 ± 0.08 a

Data show the mean ± standard deviation of three replicates from samples. Different lowercase letters indicate a significant difference at 0.05 level between different treatments.

**Table 3:** Correlation between photosynthesis and antioxidant activity of *P. heterophylla.*

	Yield	Biomass Allocation	Pn	Gs	Ci	Tr	MDA	SOD	POD	CAT	Fv/Fm	Fv′/Fm′	φPSII	qP	NPQ
Yield	1														
Biomass allocation	0.998 **	1													
Pn	0.947 *	0.944 *	1												
Gs	0.802	0.775	0.913	1											
Ci	−0.927	−0.914	−0.998 *	−0.962 *	1										
Tr	0.684	0.666	0.871	0.962 *	−0.907	1									
MDA	−0.995 **	−0.986 *	−0.942	−0.835	0.938	0.705	1								
SOD	0.880	0.909	0.842	0.548	−0.753	0.512	−0.827	1							
POD	0.906	0.925	0.940	0.727	−0.876	0.719	−0.867	0.964 *	1						
CAT	0.951 *	0.929	0.918	0.898	−0.946	0.758	−0.977 *	0.695	0.773	1					
Fv/Fm	0.808	0.781	0.912	1.000 **	−0.962 *	0.955 *	−0.842	0.548	0.724	0.906	1				
Fv′/Fm′	0.830	0.804	0.925	0.999 **	−0.971 *	0.948	−0.862	0.575	0.743	0.920	0.999 **	1			
φPSII	0.847	0.826	0.947 *	0.996 **	−0.983 *	0.957 *	−0.872	0.625	0.788	0.915	0.995 **	0.997 **	1		
qP	0.850	0.852	0.972 *	0.900	−0.954 *	0.923	−0.838	0.801	0.931	0.812	0.895	0.901	0.932	1	
NPQ	−0.863	−0.845	−0.961 *	−0.990 **	0.989 *	−0.954 *	0.884	−0.659	−0.816	−0.917	−0.989 *	−0.993 **	−0.999 **	0.964	1

** correlation is significant at the 0.01 level; * correlation is significant at the 0.05 level.

## Data Availability

All data analyzed or generated during this study are available within the manuscript and can be requested from the corresponding author.
